# Electronic Voting to Improve Morbidity and Mortality Conferences

**DOI:** 10.1007/s00268-018-4670-2

**Published:** 2018-05-16

**Authors:** Joel Zindel, Reto M. Kaderli, Manuel O. Jakob, Michel Dosch, Franziska Tschan, Daniel Candinas, Guido Beldi

**Affiliations:** 1 Department of Visceral Surgery and Medicine, Inselspital, Bern University Hospital, University of Bern, 3010 Bern, Switzerland; 2 Institute for Work and Organizational Psychology, University of Neuchatel, Neuchâtel, Switzerland

## Abstract

**Background:**

It is of major importance in clinical surgery to identify potential patterns and specific causes of complications. Therefore, morbidity and mortality meetings (M&M) are widely used to discuss and evaluate deviations from expected outcomes in order to improve surgical practice. Moreover, M&M represent an important tool for continuous medical education. In this study, we introduced an electronic voting system to assess whether anonymity during M&M could limit potential biases due to hierarchical structures or opinion leaders.

**Methods:**

This study was conducted in the surgical department of a European tertiary care center. During the study period, electronic voting was applied in 412 M&M cases and compared with a baseline of 330 conventional M&M entries. In this interrupted time series, the educational quality and participant satisfaction of the M&M were assessed using surveys before and after the introduction of electronic voting. The surveys were refined using principle component analysis. In addition, the classification of the cause of the complication was recorded.

**Results:**

The introduction of electronic voting led to a significant increase in perceived educational quality from 2.63 to 3.36 (*p* < 0.01), and the overall participant satisfaction increased from 2.6 ± 0.9 to 3.7 ± 1.2 (*p* < 0.01) on a five-point Likert scale. The frequency of voting shifted from “patient’s disease” (before 42.9, after 27.6%, *p* = 0.04) to “misadventure” (before 1.1, after 16.0%, *p* < 0.01). The voting frequencies for the causes attributed to “management” and “technical” remained constant.

**Conclusions:**

An electronic voting system in M&M meetings increases perceived educational quality and participant satisfaction.

**Electronic supplementary material:**

The online version of this article (10.1007/s00268-018-4670-2) contains supplementary material, which is available to authorized users.

## Introduction

At least half of all surgical complications are avoidable [1–3]. Morbidity and mortality meetings (M&M) provide a means to identify avoidable complications and thereby to improve surgical and medical management [4–7]. The main goal of M&M is to analyze medical incidences in order to better understand causative factors and to assess alternative decision-making. In parallel, M&M ensure continuous medical education of trainees and staff [8–11].

For a comprehensive discussion of medical errors, it is of utmost importance to ensure standardization of M&M especially within a department and ideally between institutions. Attempts to standardized M&M include root-cause analysis (RCA) which is an approach to identify the underlying cause or causes of a problem. It is designed to answer three basic questions: what happened, why did it happen, and what can be done to prevent it from happening again [12]. Another attempt to generate standardized safety communications in M&Ms is the SBAR (*Situation*, *Background*, *Assessment*, and *Recommendations*) which was originally introduced in high-risk industries from where it was adopted for use in medicine [13]. However, the adoption of RCA and SBAR in medicine, and in particular surgery, is hampered by specific limitations such as underreporting [14–16], lack of sufficient information, inadequate presentation [17, 18], and especially by the potential for differences due to hierarchical structures [17–20].

Therefore, to specifically address challenges in the medical field, M&M frameworks should address the following three main areas:

1. Standardized identification, reporting, and presentation of cases. Self-reporting of cases by individual surgeons should be circumvented as this approach is prone to underreporting: surgeons may avoid discussions of complications of their own patients [14, 19, 20]. Standardized methods to identify adverse events and preventable deaths have been described in the literature. Various systems have been implemented in the surgical environment such as the global trigger tool [21], or reporting systems that include external validation, such as the national American College of Surgeons-National Surgical Quality Improvement program (ACS-NSQIP). Trigger tools, registries, or other quality assurance initiatives, have been shown to significantly increase the rate of complications detected [13–16, 21].
2. Objective and standardized analysis and discussion of complications. The discussion should classify complications into causative categories. Despite the lack of a consensus on a classification, the distinction between preventable and unpreventable is widely accepted [4, 8, 22–26]. Preventable causes include technical aspects, decision-making, and team factors. Potentially unpreventable causes include patient-related factors, simple bad luck and unknown reasons for a complication [16, 27]. However, such classifications do not reflect that the majority of medical errors are based on multifactorial causes [28]. To classify complications, an open and free discussion, that allows all participants to contribute, is important. However, clinical or hierarchical opinion leaders may bias such an open discussion [4, 19, 25, 29–31].
3. Translation: Implicit or explicit translation of what was learned into daily clinical practice should be attempted by formulating general or specific recommendations [10, 13]. This element is reflected by the major role M&M play in educational initiatives. In the USA, M&M have become a required part of training for both surgical and medical residents and many countries, including the UK, have followed suit [7, 32].

The aim of this initiative was to improve discussion, satisfaction, and education for participants attending M&M. Therefore, an electronic voting system was introduced to encourage free decision-making and to allow the simultaneous identification and assessment of the relative importance of multiple causes of complications. Electronic voting was embedded into a novel framework that was incorporated into the existing SBAR framework. It includes standardized case selection, standardized case presentation and whenever possible a recommendation.

Within the present prospective cohort study, we explored the impact of electronic voting on participant satisfaction and educational quality.

## Materials and methods

This study was performed as an interrupted time series design in a European tertiary care center. Baseline measurements were assessed before and compared to measurements after the introduction of electronic voting.

### Baseline

Before the start of the study, identification and selection of patients was based on a voluntary reporting system. At M&M, case presentation was done by an intern familiar with the case, followed by a free discussion, led by a senior consultant. After the discussion, the complications were categorized according to the usual classification of the institution into the categories “technical,” “management,” “patient’s disease,” or “misadventure.” Votes for classification of complications were recorded electronically in a database. During the baseline period, unsystematic iterative observations by professional work psychologists were performed in order to identify potential bias. These observations were the basis for the design of the novel framework that includes electronic voting.

### Intervention

The novel framework and electronic voting was introduced on December 5, 2014. After a test period of 4 months, the electronic voting was definitively installed on April 1, 2015, and was formally validated within the current study.

**Fig. 1:**
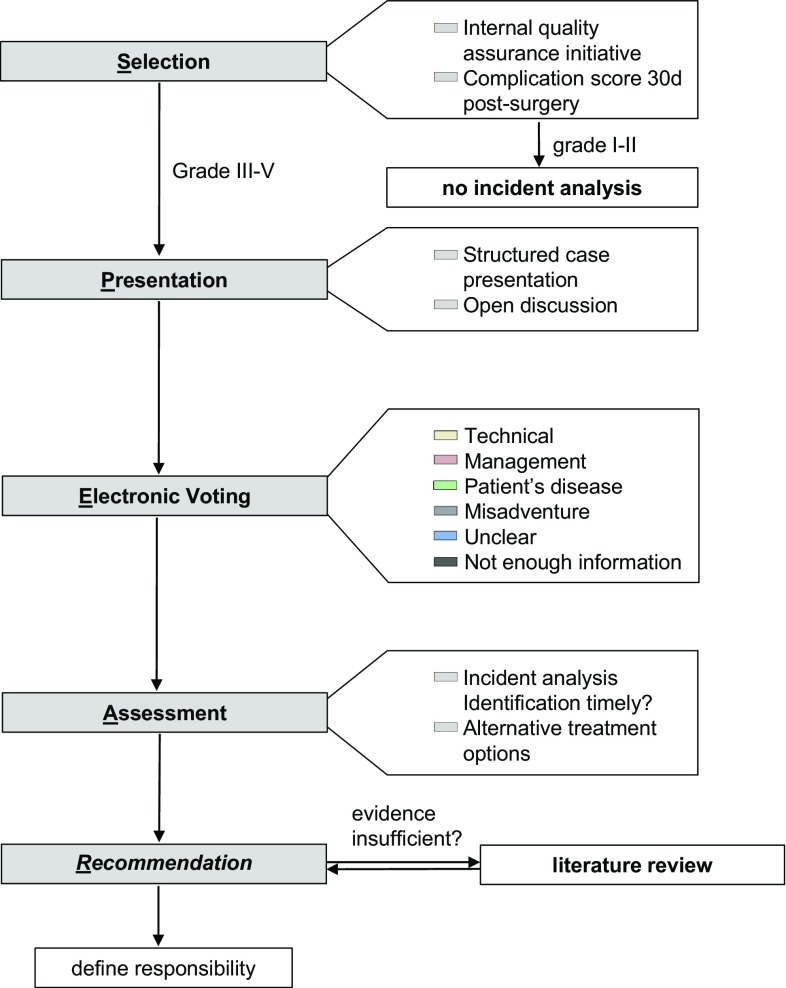
*SPEAR* (selection, presentation, electronic voting, recommendation) framework

The adaptation of the existing SBAR framework was done in order to address the needs of the healthcare industry. The adapted acronym is SPEAR: Selection, Presentation, Electronic Voting, Assessment, Recommendation, and it includes the following elements (Fig. 1):

### Selection

Standardized identification and selection of cases was implemented based on an existing quality assurance initiative. All surgical procedures were continuously listed by external study nurses. At least 30 days after surgery, all patient records were screened for any deterioration of normal recovery by an independent (i.e., non-treating) physician. This physician did not receive any specific training. The selection process was evaluated and certified within a process audit (Deutsche Krebsgesellschaft e.V. OnkoZert, ISO 9001, registry number: FAD-Z333 V). All deteriorations were graded according to Clavien–Dindo Complication Score [33] and entered into a quality control database. All complications scoring three or higher were selected to be discussed at the next M&M. Lower graded complications were discussed only upon request from the responsible surgeon.

### Presentation

A specific graphical representation was used in order to clearly define and visualize the entire workflow (Fig. 1). Structured case presentation was done by the treating physician using a standardized Microsoft^®^ PowerPoint™ 2010 template as previously described [18]. All presenting physicians were trained in the novel workflow for 1 h by the investigators. The presentations were delivered by the lowest ranking intern of the treating team.

### Electronic voting and assessment

The electronic voting is the main novelty of the framework. In order to reduce the bias introduced by clinical or hierarchical opinion leaders, all participants classified potential causes of the complication by an anonymous electronic vote at the end of the discussion. For electronic voting, a remote control (IML Click^®^, Lumi Technologies LTD, Liphook, UK) was used. Single or multiple choice answers for the classification categories: “technical”, “management”, “patient’s disease”, “misadventure”, “unclear”, “not enough information”. These categories were used according to the following definitions:

- *Technical* Attributed to the application of improper surgical technique.
- *Management* Occurred due to potential errors in the perioperative management of the patient.
- *Patient’s disease* Due to an underlying disease of the patient. Failures in management should be ruled out.
- *Misadventure* Due to a random event that cannot be possibly controlled for by proper technique and/or management.
- *Unclear* Information available is precise but ambiguous.
- *Not enough information* Information available is not precise enough/vague. Further information should be obtained whenever possible.

The voting results were displayed as percentages for each possible answer on-screen using Microsoft^®^ PowerPoint™ 2010.

### Recommendation

According to the framework (Fig. 1) “alternative treatment options” were considered. If the evidence present was sufficient, a recommendation was directly formulated. If not, a short review of the literature and a choice of recommendations were prepared for the next M&M.

### Outcome measurements

#### Patients

All patients operated between January 1, 2013, and December 31, 2016, were included. Number of operations performed per year and number of identified complications were registered. Patient data were extracted retrospectively after anonymization from the M&M database.

#### Survey

A survey (adapted from Bechtold ML et al. [34]) was performed to assess satisfaction, learning effect, perceived personal integration and participation at the M&M conference (Supplementary Table 1). The questionnaire included five categories. 1. “demographic questions” (3 items), 2. “institutional error culture” (12 items), 3. “goals and consequences of M&M” (10 items) and 4. “individual perceived benefit of M&M” (20 items). Category 5 was the stand-alone question of “overall satisfaction”.

**Table 1:** Participant demographics

	First survey	Second survey
Number of participants	40	33
Position *n* (%)		
Staff surgeons	5 (12.5)	3 (9.1)
Fellows	12 (30.0)	11 (33.3)
Residents	18 (45.0)	14 (42.4)
Interns	5 (12.5)	5 (15.2)
Surgical experience in years (±SD^a^)*	3.39 (1.75)	4.00 (1.66)
Gender female (%)	20 (50.0)	15 (45.5)

*Only applicable for residents

^a^*SD* standard deviation

All non-demographic items were answered on a five-point Likert scale with 1 being the lowest and 5 being the highest value. The survey was performed before and after the introduction of the novel framework. The first survey took place between September 1 and November 30, 2014. The second survey was conducted between February 22 and March 31, 2015 after training of the staff in the novel framework. The anonymous, electronic survey was sent to all physicians and students participating in the M&M. Primary non-responders were reminded by additional emails and direct contact.

**Fig. 2:**
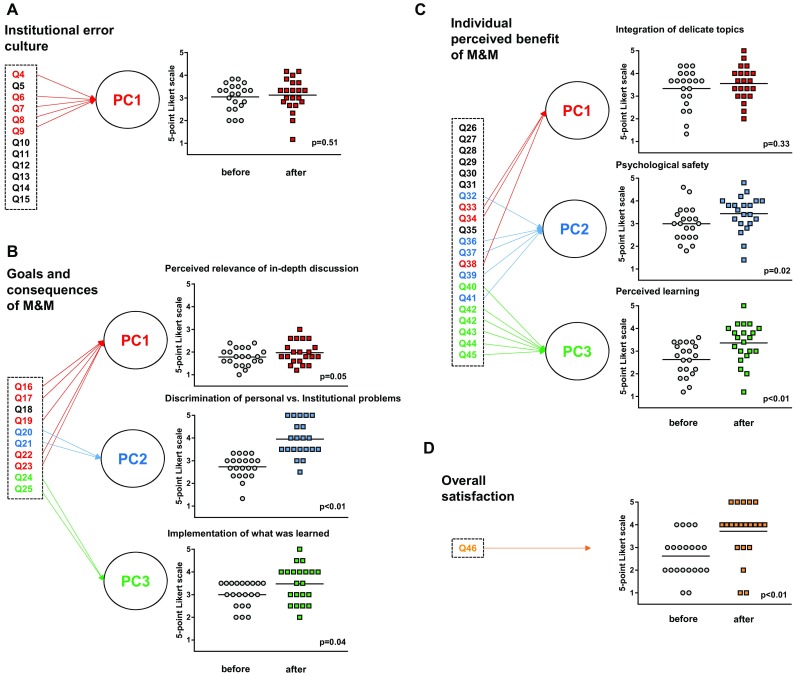
Survey results. Principle component analysis (PCA) of the three different survey categories “institutional error culture” (**a**), “goals and consequences of M&M” (**b**), “individual perceived benefit” (**c**). Items are mathematically reduced to individual principle components (PC). Category “overall satisfaction” comprised only the question of overall satisfaction. **a** PCA of “institutional error culture” resulted in one single PC. **b** PCA of “goals and consequences of M&M” resulted in three PC. PC1 contains items on the relevance of the in-depth discussion. PC2 contains two items assessing how well the M&M is able to discriminate personal from institutional causes of medical complications. PC3 assesses how recommendations are implemented into daily practice. **c** PCA of “individual perceived benefit of M&M” resulted in three PC. PC1 contains items on the integration of delicate topics. PC2 contains items on psychological safety (such as emotional comfort) during M&M. PC3 contains items assessing educational effectiveness of M&M. **d** Overall satisfaction is represented here as a stand-alone single item question without PCA

Principal component analysis (PCA) was carried out to refine the questionnaire. This technique is used to capture the multi-dimensionality of multiple-item questionnaires. Thereby, multiple items (questions) are reduced to a small number of principle components (PC). These PC then describe most of the variability in the data and are more comprehensive to report. In this process, items may also be dropped from analysis if they are redundant and do not contribute to describing total variability of the data. To test the results, measure of sampling adequacy and Cronbach’s alpha were used to identify whether the principle components found are robust (a Cronbach’s alpha >0.65 was considered acceptable) [35]. For a detailed description, we refer the reader to supplementary appendix. The left side of Fig. 2 graphically explains the process of principle component analysis: PCA of the category “institutional error culture” results in a single PC (Fig. 2a). PCA of the categories “goals and consequences of M&M” and “individual perceived benefit of M&M” result in three PC each (Fig. 2b, c). In addition, the question no. 46 assessing overall satisfaction was reported as single item (Fig. 2d).

To be able to compare surveys of the same participants before and after the intervention, participants entered a confidential code, used to match the surveys. Independent-sample test between participants answering only one survey revealed no significant difference (*p* = 0.88). Only participants who completed both surveys were included in the statistical analysis.

#### Voting results

Voting results and complications of M&Ms have been prospectively entered since January 2010 within a quality control database. The voting results of the last year before the introduction of electronic voting (January 1, 2013, until December 31, 2013) were compared to the first year following the introduction of electronic voting (January 1, 2016, until December 31, 2016).

#### Statistics

Statistical analysis was performed using SPSS (IBM Corp. Released 2012. IBM SPSS Statistics for Windows, Version 21.0. Armonk, NY: IBM Corp.). *P* values were two-tailed, with *p* < 0.05 determining statistical significance. Except for demographic data, all questionnaire items were based on a five-point Likert scale. Data are presented as mean (±standard deviation). Statistical significance of principle components (five-point Likert scale data) was assessed using paired *t* tests [36]. Kruskal–Wallis H tests were used to analyze the data from the voting results before and after the introduction of the M&M framework.

## Results

### Survey results

The final response rate for each survey was 87% (40/46) and 94% (33/35) (Table 1).

### Patients

During the study period electronic voting was applied in 1147 M&M cases. As a baseline comparison, 330 entries of conventionally held M&M entries were extracted.

### Institutional error culture

Institutional error culture (PC1) remained constant at 3.0 (±0.59) before and 3.1 (±0.73; *p* = 0.51) after the implementation of electronic voting (Fig. 2a). The baseline error culture of the department is shown in Supplementary Fig. 1.

### Goals and consequences of M&M

All principle components of the survey category “goals and consequences of M&M” were significantly increased (Fig. 2b): PC1 assessed the relevance of the in-depth discussion and increased from 1.78 (±0.38) to 1.97 (±0.49) (*p* = 0.045). PC2 assessed the ability of the M&M to discriminate personal from institutional problems and increased from 2.73 (±0.50) to 3.95 (±0.76) (*p* < 0.01). PC3 assessed the implementation of recommendations into daily practice and increased from 3.00 (±0.55) to 3.48 (±0.81) (*p* = 0.04).

### Individual perceived benefits of M&M

After introduction of electronic voting, the perceived personal benefits of M&M increased significantly (Fig. 2c). PC1, which assessed the integration of delicate topics, was not significantly different (before 3.33 (±0.85), after 3.56 (±0.75), *p* = 0.33). PC2 assessed psychological safety (e.g., “I feel comfortable during M&M”) and increased significantly from 2.99 (±0.74) to 3.44 (±0.80) (*p* = 0.02). PC3 assessed the educational effectiveness and increased significantly from 2.63 (±0.71) to 3.36 (±0.88) (*p* < 0.01).

Overall satisfaction increased from 2.6 to 3.7 (*p* < 0.01) (Fig. 2d).

### Votes for causes of complications

**Fig. 3:**
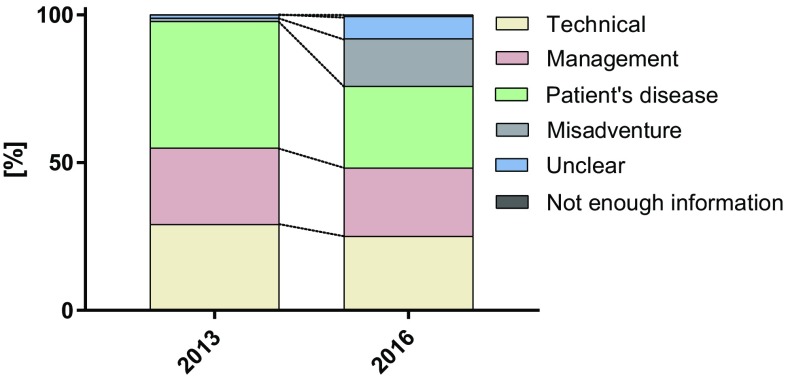
Votes for causes of complications. The distribution (frequency) of the votes for the different categories before and after the introduction of the novel framework is shown

The number of votes provided for the cause of complication before and after the introduction of electronic voting was compared. Figure 3 shows the distribution (frequency) of the votes for the different categories. One or more votes were possible. The percentages of votes for the causes ‘technical’ and ‘management’ remained unchanged after the intervention (*p* = 0.40). There was a significant decrease in the most voted for category “patient’s disease” (before 42.9% vs. after 27.6%, *p* = 0.04), and an increase in the votes for the two categories “misadventure” (before 1.1% vs. after 16.1%, *p* < 0.01) and “unclear” (before 1.1% vs. after 7.5%, *p* = 0.03).

## Discussion

M&M are a valuable and simple tool for quality control and education. In this study, we have designed and implemented an electronic voting system within a novel M&M framework. We observed a significant increase in the perceived benefit of M&M by the participants, particularly with respect to its educational effectiveness. The benefit of this novel framework may be a consequence of electronic voting and its effect in reducing hierarchical bias therefore empowering the whole audience. In the proposed SPEAR framework, electronic voting was performed after the case was discussed, therefore a remaining influence of opinion leaders cannot be ruled out. In future validation studies of this framework, voting could take place before the discussion of the case and after to infer on the influence by senior/opinion leader staff.

In addition, overall satisfaction “goal and consequences of M&M” were significantly increased, indicating an increase in validity and acceptance of the M&M’s conclusions.

The selection of complications has been highly standardized by the introduction of the novel M&M framework and electronic voting. The introduction of electronic voting was associated with an increase in the scope of the interpretation of complications. The category “misadventure” was chosen more frequently potentially revealing an uncertainty of the causes. This supports the hypothesis of a potential bias because of hierarchical structures before the introduction of electronic voting in which judgment was more dependent on the opinion of the leader of the discussion. However, the number of complications judged to be potentially preventable, such as “technical” or “management” errors, remained constant at around 50%. This is in line with the often-cited estimation that half of medical complications are preventable [1–3].

No change in institutional error culture was observed, this being potentially based on a well-accepted error culture within our department at the beginning of this study. Thus, the current study reveals that improvement of M&M is possible by providing key structures without fundamental change in the underlying error culture. Electronic voting seems to be the most important reason for the results of this study. In this study a voting tool was used that needs the purchase of additional hardware (iml click^®^, Lumi insight). The accompanying software ViewPoint is free. As simple and effective alternative tools, existing smartphone applications could be used. Several Web-based smartphone applications are provided free of charge and have been validated for academic use [37]. Another interesting opportunity may lie in a combination of electronic voting along with interactive multi-site video teleconference M&M as recently proposed [38].

## Conclusions

The introduction of electronic voting was associated with a significant increase in the educational quality of M&M.

## Electronic supplementary material

Below is the link to the electronic supplementary material.

Supplementary material 1 (DOCX 11 kb)

Supplementary Fig. 1 Institutional error culture. The distribution of all answers on a five-point-Likert scale for the second category, institutional error culture, are shown (PDF 5 kb)

Supplementary Table 1 Survey questions and categories are shown (DOCX 16 kb)

## Abbreviation

M&M: Morbidity and mortality
