# Supplementary material for: Electronic Voting to Improve Morbidity and Mortality Conferences
Source: World J Surg. 2018 May 16;42(11):3474–81. doi: 10.1007/s00268-018-4670-2 (PMC6182754; doi:10.1007/s00268-018-4670-2)
Supplement: Supplementary file 1 — Supplementary material 1 (DOCX 11 kb) [file 268_2018_4670_MOESM1_ESM.docx]

## Supplementary appendix

Additional methods

Principal components analysis (PCA) was carried out to refine the questionnaire. This approach is often used by psychologists to reduce the number of dimensions (i.e. number of questions) of a questionnaire to report without losing information. These principle components (PCs) reduce complexity of the results into a few components and in paralle increases the robustness of the questionnaire. As the answers of differently phrased questions with identical or opposite meaning are averaged. This analysis is done separately for each part of a questionnaire called instrument. The Bartlett and Kaiser-Meyer-Olkin (KMO) tests were used to assess whether the underlying data structure was suited for PCA. Data with a significant Bartlett test (p <0.05) and a KMO > 0.5 are generally considered to be suited for PCA.

The instrument *institutional error culture* (items Q08-Q20) revealed a single factorial solution. This showed a significant Bartlett-Test (Chi-Square(120) = 255.30, p<0.001) and a good Kaiser-Meyer-Olkin (KMO) Measure of Sampling Adequacy, KMO = 0.71). The resulting model was able to explain 64.0% of total variance, with one principal component (Eigenwert=3.5).

PCA of the instrument *goals and consequences of M&M* showed a borderline measure of sample adequacy (KMO=0.52). Bartlett-Test was significant however (Chi-Square(120) = 168.2, p<0-001), and PCA was carried out. The extraction showed four factors with an Eigenvalue > 1.0, but according to the Screeplot and theoretical considerations and as well for better interpretability of results, a three-factor solution was chosen, explaining 56.7% of total variance: *first* Perceived relevance of in-depth discussion (Eigenwert 2.28), *second* discrimination of personal vs. institutional problems (Eigenwert 1.87) and *third* implementation of what was learned (Eigenwert 1.52).

PCA of the instrument *individual perceived benefit of M&M* showed a favorable measure of sample adequacy (KMO 0.80, Bartlett-Test Chi Square (120) = 238.34, p<0.001). The extraction revealed four factors with an Eigenvalue > 1.0. Component four however was very closely to 1 (Eigenwert=1.04) and interpretability also favored a three-factor solution. The fitted model was able to explain 61.75% of total variance. The factors were: first integration of delicate topics (Eigenwert 1.31), second psychological safety (Eigenwert 1.06) and third perceived learning (Eigenwert 5.72).
