# Supplementary figures and images for: Electronic Voting to Improve Morbidity and Mortality Conferences
Source: World J Surg. 2018 May 16;42(11):3474–81. doi: 10.1007/s00268-018-4670-2 (PMC6182754; doi:10.1007/s00268-018-4670-2)

## General error culture

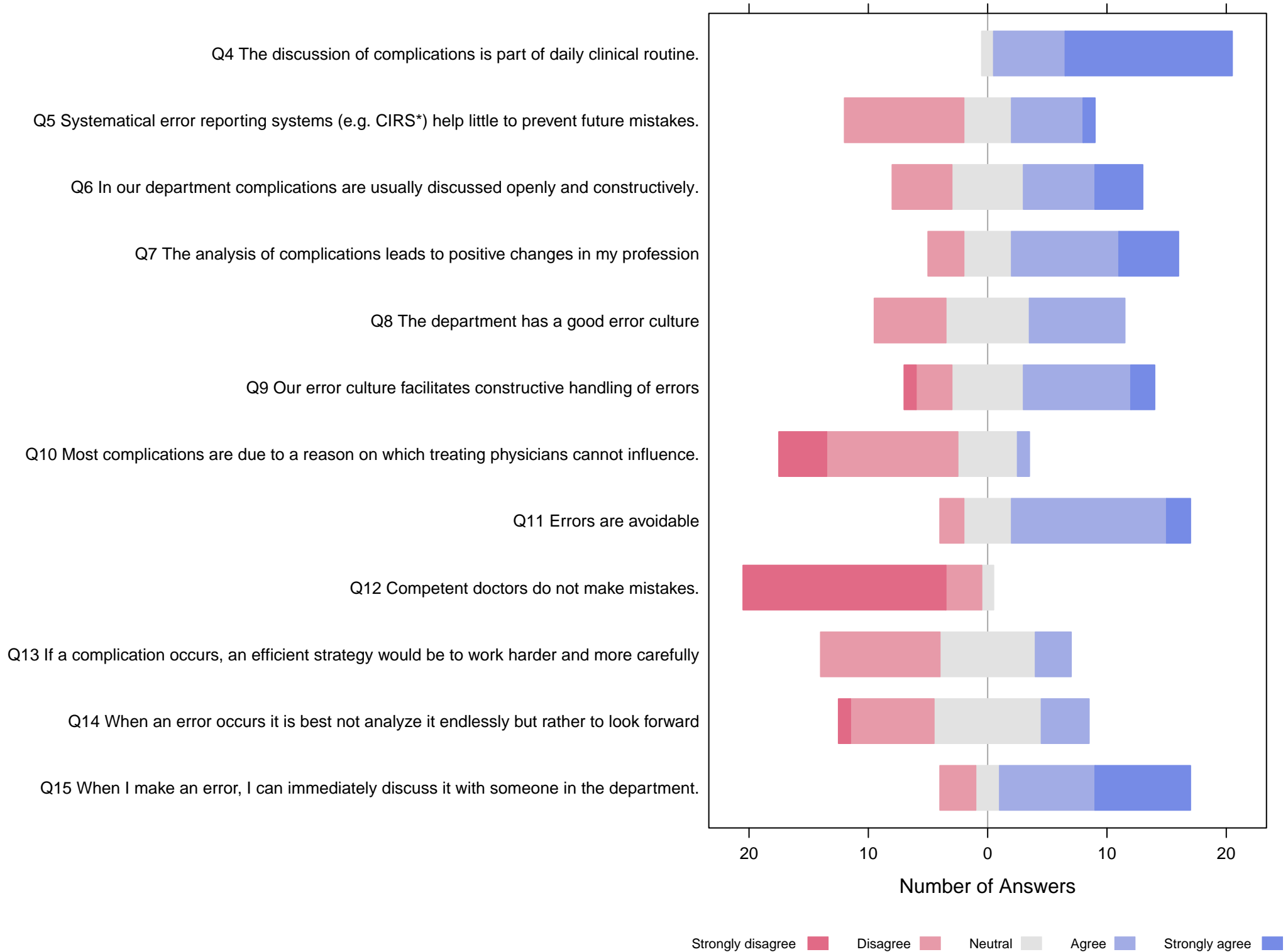

Supplement: Supplementary file 2 — Institutional error culture. The distribution of all answers on a five-point-Likert scale for the second category, institutional error culture, are shown (PDF 5 kb) [file 268_2018_4670_MOESM2_ESM.pdf]
