# Supplementary material for: Electronic Voting to Improve Morbidity and Mortality Conferences
Source: World J Surg. 2018 May 16;42(11):3474–81. doi: 10.1007/s00268-018-4670-2 (PMC6182754; doi:10.1007/s00268-018-4670-2)
Supplement: Supplementary file 3 — Survey questions and categories are shown (DOCX 16 kb) [file 268_2018_4670_MOESM3_ESM.docx]

| **Demographics** | | |
| --- | --- | --- |
| Q1 | Please indicate your current position within the clinic | 1 Intern  🡪 skip Q2  2 Resident  3 Fellow / attending  🡪 skip Q2  4 Senior consultant  🡪 skip Q2 |
| Q2 | How many years of surgical training do you have (residents only) | 1 1 year  2 2 years  3 3 years  4 4 years  2 5 years  3 6 years  4 > or equal 7 years |
| Q3 | Gender | male / female |
| **Institutional error culture** | | |
| Q4 | The discussion of complications is part of daily clinical routine. | |
| Q5 | Systematical error reporting systems (e.g. CIRS*) help little to prevent future mistakes. | |
| Q6 | In our department complications are usually discussed openly and constructively. | |
| Q7 | The analysis of complications leads to positive changes in my profession. | |
| Q8 | The department has a good error culture. | |
| Q9 | Our error culture facilitates constructive handling of errors. | |
| Q10 | Most complications are due to a reason on which treating physicians cannot influence. | |
| Q11 | Errors are avoidable. | |
| Q12 | Competent doctors do not make mistakes. | |
| Q13 | If a complication occurs, an efficient strategy would be to work harder and more carefully. | |
| Q14 | When an error occurs it is best not to analyze it endlessly but rather to look forward. | |
| Q15 | When I make an error, I can immediately discuss it with someone in the department. | |
| **Goals and consequences of M&M** | | |
| Q16 | An important goal of M&M is the identification of weaknesses in knowledge and skill of the treating physician. | |
| Q17 | An important goal of M&M is the discussion of intraoperative decisions and strategies. | |
| Q18 | An important goal of M&M is the discussion of technical aspects of surgery. | |
| Q19 | An important goal of M&M is the discussion of problems in communication. | |
| Q20 | An important goal of M&M is the discussion of personal weaknesses. | |
| Q21 | An effective M&M should focus on general causes of medical complications. | |
| Q22 | Most of the medical complications discussed at M&M are caused by a single person. | |
| Q23 | An effective M&M should focus on how the treating physician should have behaved. | |
| Q24 | Suggestions deduced from M&M are usually implemented. | |
| Q25 | Strategies or guidelines deduced from M&M are frequently disregarded. | |
| **Individual perceived benefits of M&M** | | |
| Q26 | I do not participate in M&M whenever possible. | |
| Q27 | It would be good if experienced surgeons could participate more often in M&M. | |
| Q28 | I feel comfortable during M&M. | |
| Q29 | It is helpful for me when my patients’ complications are discussed at M&M. | |
| Q30 | The topics discussed at M&M help me during my daily work. | |
| Q31 | During the presentation of cases, the treating physician is often exposed. | |
| Q32 | I feel tense when one of my patients is presented. | |
| Q33 | I regularly participate in the discussion. | |
| Q34 | At M&M we avoid certain topics. | |
| Q35 | The truly important discussions are held after M&M. | |
| Q36 | I feel comfortable during analysis of a medical complication. | |
| Q37 | At M&M one can readily approach sensitive topics. | |
| Q38 | Discussions at M&M are often boring. | |
| Q39 | I sometimes get annoyed with the way the discussion is held. | |
| Q40 | Cause and Consequences of medical complications are deduced concisely. | |
| Q41 | Personal errors are discussed concisely and openly. | |
| Q42 | If measures are discussed, they are formulated concisely. | |
| Q43 | The body of evidence is usually sufficient to discuss the cases. | |
| Q44 | M&M is educational in its current format. | |
| Q45 | M&M is efficient in its current format. | |
| **Overall satisfaction** | | |
| Q46 | How satisfied are you with the M&M in its current format. | |
